# Association between myosin heavy chain protein isoforms and intramuscular anabolic signaling following resistance exercise in trained men

**DOI:** 10.14814/phy2.12268

**Published:** 2015-01-27

**Authors:** Adam M. Gonzalez, Jay R. Hoffman, Jeremy R. Townsend, Adam R. Jajtner, Adam J. Wells, Kyle S. Beyer, Darryn S. Willoughby, Leonardo P. Oliveira, David H. Fukuda, Maren S. Fragala, Jeffrey R. Stout

**Affiliations:** Institute of Exercise Physiology and Wellness, Sport and Exercise Science, University of Central Florida, Orlando, Florida, USA; Exercise Nutrition and Resistance Training Research Unit, Baylor University, Waco, Texas, USA; Department of Internal Medicine, College of Medicine, University of Central Florida, Orlando, Florida, USA

**Keywords:** Anabolic signaling, mTOR pathway, mTORC1, myosin heavy chain mRNA expression, p70S6k

## Abstract

Resistance exercise stimulates an increase in muscle protein synthesis regulated by intracellular anabolic signaling molecules in a mammalian/mechanistic target of rapamycin (mTOR)‐dependent pathway. The purpose of this study was to investigate acute anabolic signaling responses in experienced, resistance‐trained men, and to examine the association between myosin heavy chain (MHC) isoform composition and the magnitude of anabolic signaling. Eight resistance‐trained men (24.9 ± 4.3 years; 91.2 ± 12.4 kg; 176.7 ± 8.0 cm; 13.3 ± 3.9 body fat %) performed a whole body, high‐volume resistance exercise protocol (REX) and a control protocol (CTL) in a balanced, randomized order. Participants were provided a standardized breakfast, recovery drink, and meal during each protocol. Fine needle muscle biopsies were completed at baseline (BL), 2 h (2H) and 6 h post‐exercise (6H). BL biopsies were analyzed for MHC isoform composition. Phosphorylation of proteins specific to the Akt/mTOR signaling pathway and MHC mRNA expression was quantified. Phosphorylation of p70S6k was significantly greater in REX compared to CTL at 2H (*P* = 0.04). MHC mRNA expression and other targets in the Akt/mTOR pathway were not significantly influenced by REX. The percentage of type IIX isoform was inversely correlated (*P* < 0.05) with type I and type IIA MHC mRNA expression (*r* = −0.69 to −0.93). Maximal strength was also observed to be inversely correlated (*P* < 0.05) with Type I and Type IIA MHC mRNA expression (*r* = −0.75 to −0.77) and p70S6k phosphorylation (*r* = −0.75). Results indicate that activation of p70S6k occurs within 2‐h following REX in experienced, resistance‐trained men. Further, results also suggest that highly trained, stronger individuals have an attenuated acute anabolic response.

## Abstract (precis)

Resistance exercise stimulates an increase in muscle protein synthesis regulated by intracellular anabolic signaling molecules in a mammalian/mechanistic target of rapamycin (mTOR)‐dependent pathway. Results indicate that activation of p70S6k occurs within 2‐h following REX in experienced, resistance‐trained men. Further, results also suggest that highly trained, stronger individuals have an attenuated acute anabolic response.

## Introduction

The anabolic response following resistance exercise appears to be highly variable between individuals (Hubal et al. 2005; Coffey et al. 2006; Bamman et al. 2007; Davidsen et al. 2011). There are a number of factors that influence the muscle remodeling process following resistance exercise. These include nutritional intake, training status, and genetic predisposition. At the cellular level, adaptations within skeletal tissue appear to be the result of the cumulative effects of transient changes in gene expression following acute bouts of exercise (Coffey and Hawley 2007). Resistance exercise appears to stimulate an increase in muscle protein synthesis for up to 48 h post‐exercise (Chesley et al. 1992; Yarasheski et al. 1993; MacDougall et al. 1995; Phillips et al. 1997). This appears to be regulated by intracellular anabolic signaling molecules through a mammalian/mechanistic target of rapamycin (mTOR)‐dependent pathway (Welle et al. 1999; Baar et al. 2006). Resistance exercise rapidly activates the signaling cascade through phosphorylation of upstream (i.e., Akt) and downstream (i.e., p70S6k, RPS6) effectors of the mTOR complex 1 (mTORC1) (Coffey et al. 2006; Koopman et al. 2006; Camera et al. 2010; Farnfield et al. 2011). Regulation of mRNA translation initiation through mTORC1 appears to be the primary mediator of muscle protein synthesis (Hornberger et al. 2006; Drummond et al. 2009; Goodman 2014). Phosphorylation of p70S6 kinase (p70S6k) modulates mRNA translation initiation by regulating the binding of mRNA to the 40S ribosomal subunit (Kimball et al. 2002). Furthermore, the magnitude of p70S6k phosphorylation has been shown to be a predictor of myofibrillar protein synthesis rates (Kumar et al. 2009), potentially leading to increased muscle cross‐sectional area (Baar and Esser 1999; Terzis et al. 2008; Mayhew et al. 2009; Mitchell et al. 2013).

Resistance training stimulates increases in muscle fiber size (Staron et al. 1990, 1991; McCall et al. 1996; Hikida et al. 2000; Campos et al. 2002; West et al. 2010b; Mitchell et al. 2012), but the magnitude of muscle fiber hypertrophy tends to be greater in type II compared to type I fibers (Tesch 1987; Staron et al. 1990; Adams and Bamman 2012). These changes are likely mediated by pretranslational mechanisms including upregulation of mRNA expression of myosin heavy chain (MHC) isoforms in skeletal muscle (Caiozzo et al. 1996; Willoughby and Rosene 2001; Willoughby and Nelson 2002; Wilborn et al. 2009). Significant increases in mRNA expression of type I, IIA, and IIX MHC isoforms have been observed six hours following a single bout of resistance exercise in recreationally active men (Willoughby and Nelson 2002). Changes in muscle cross‐sectional area resulting from resistance training are attributed to the synthesis of myofibrillar protein content (McCall et al. 1999; Willoughby and Rosene 2001). An upregulation of MHC mRNA content presumably increases the rate of translational encoding for the respective MHC protein (Willoughby and Rosene 2001).

The evidence supporting the greater hypertrophy potential of type II compared to type I muscle fibers subsequent to a resistance exercise stimulus suggests that the phenotypic composition of skeletal muscle may impact the genes regulating translation initiation signaling and MHC mRNA expression. In studies examining a murine model, resistance exercise was reported to induce Akt and p70S6k phosphorylation to a greater extent in the predominantly fast‐twitch extensor digitorum longus muscle compared to the slow‐twitch soleus (Baar and Esser 1999; Sakamoto et al. 2003), and mTOR phosphorylation has shown to be specific to fibers predominantly expressing the MHC IIA isoform (Parkington et al. 2003). A fiber type‐specific anabolic signaling response has also been reported in human skeletal muscle (Trappe et al. 2004; Carroll et al. 2005). Using immunohistochemical techniques, Koopman et al. (2006) showed that phosphorylation of p70S6k was increased following resistance exercise in both type I and type II fibers, but p70S6k phosphorylation was significantly greater in the type II fibers of young untrained men. Fiber type‐dependent mTOR phosphorylation may provide a molecular basis by which some fiber types are more sensitive for resistance exercise‐induced hypertrophy. However, whether this same effect is seen in the experienced, resistance‐trained individual is unknown.

Thus, the primary purpose of this present study was to investigate the acute anabolic signaling response following a bout of resistance exercise in experienced, resistance‐trained men utilizing a high‐volume, whole body protocol typically used by athletes focusing on maximizing muscle hypertrophy. A secondary purpose was to examine the association between MHC isoform composition and the magnitude of anabolic signaling.

## Materials and Methods

### Participants

Eight resistance‐trained men (24.9 ± 4.3 years; 91.2 ± 12.4 kg; 176.7 ± 8.0 cm; 13.3 ± 3.9 body fat %) were recruited to participate in this randomized, cross‐over design research study. Strict recruitment criteria were implemented to increase homogeneity of the sample. Inclusion criteria required participants to be between the ages of 18 and 35 years, a minimum of 1 year of resistance training experience, and the ability to squat a weight equivalent to their body mass. Participants had 8.0 ± 4.9 years of resistance training experience with an average maximum barbell squat of 179.5 ± 45.3 kg and an average maximum barbell bench press of 128.4 ± 27.6 kg. All participants were free of any physical limitations that may affect performance. Additionally, all participants were free of any medications and performance‐enhancing drugs, as determined by a health and activity questionnaire. Following an explanation of all procedures, risks, and benefits, each participant provided his informed consent prior to participation in this study. The research protocol was approved by the University's Institutional Review Board prior to participant enrollment.

### Study protocol

Participants reported to the Human Performance Laboratory (HPL) on three separate occasions. On the first visit, anthropometric assessments including height, body mass, and body fat percentage were measured. Body fat percentage was assessed via skinfold analysis using a 4‐site skinfold test (Jackson and Pollock 1978). Participants were then tested for maximal strength [one repetition maximum (1‐RM)] on all lifts involved in the exercise protocol. Prior to maximal strength testing, participants performed a standardized warm‐up consisting of 5 min on a cycle ergometer against a light resistance, 10 body weight squats, 10 body weight walking lunges, 10 dynamic walking hamstring stretches, and 10 dynamic walking quadriceps stretches. The 1‐RM tests for the barbell back squat and barbell bench press were performed using methods previously described by Hoffman (2006). Briefly, each participant performed two warm‐up sets using a resistance of approximately 40–60% and 60–80% of his perceived maximum, respectively. For each exercise, 3–4 subsequent trials were performed to determine the 1‐RM. A 3–5 min rest period was provided between each trial. Trials not meeting the range of motion criteria for each exercise or where proper technique was not used were discarded. For all other exercises, the 1‐RM was assessed using a prediction formula based on the number of repetitions performed to fatigue using a given weight (Brzycki 1993).

On the morning of each trial, participants reported to the HPL following a 10‐h overnight fast and having refrained from all forms of moderate to vigorous exercise for the previous 72 h. The experimental trials consisted of a resistance exercise protocol (REX) and a control protocol (CTL) performed in a balanced, randomized order. Each experimental trial was separated by a minimum of 1 week to ensure adequate recovery.

During REX, participants performed the standardized warm‐up routine described above, followed by the resistance exercise protocol depicted in Table 1. A high‐volume, whole body resistance exercise protocol was employed to simulate what one may see being used by an athlete focusing on muscle hypertrophy (Kraemer et al. 2002). Each exercise was performed for four sets of 8–10 repetitions. The rest interval between each set and between all exercises was 60 s. For each exercise the initial load was 70% of the participant's 1‐RM. If the participant was unable to complete eight repetitions, spotters provided assistance until the subject completed the remaining repetitions. Subsequently, the load for the next set was adjusted so that participants were able to perform 8–10 repetitions. During CTL, participants sat comfortably in the HPL for the equivalent time required to complete the exercise protocol.

**Table 1.** Resistance Exercise Protocol. Each exercise was performed for four sets of 8–10 repetitions. The rest interval between each set and between all exercises was 60 s. For each exercise, the initial load was 70% of the participant's one repetition maximum (1‐RM). If the participant was unable to complete the desired number of repetitions, spotters provided assistance until the subject completes the remaining repetitions. Subsequently, the loads were adjusted so that participants were able to perform 8–10 repetitions for each set

Exercise	Sets × Repetitions	Intensity Load
Barbell Back Squat	4 × 8−10	70% 1‐RM
Barbell Bench Press	4 × 8−10	70% 1‐RM
Leg Press	4 × 8−10	70% 1‐RM
Barbell Seated Shoulder Press	4 × 8−10	70% 1‐RM
Barbell Bent‐Over Row	4 × 8−10	70% 1‐RM
Barbell Upright Row	4 × 8−10	70% 1‐RM
Barbell Bicep Curl	4 × 8−10	70% 1‐RM

Fine needle muscle biopsies were performed at baseline (BL), 2 h post‐exercise (2H), and 6 h post‐exercise (6H). All biopsies were taken at the same time of day to avoid diurnal variations. To control for diet, participants were provided a standardized breakfast (42 g carbohydrate; 10 g protein; 1.5 g fat) following the BL biopsy. Participants were also provided a recovery drink immediately following exercise (49 g carbohydrates; 26 g protein; 2 g fat) and a small meal following the 2H biopsy (61 g carbohydrate; 18 g protein; 10 g fat). Meals were provided at the same time of day during each experimental trial. Participants were permitted to drink water ad libitum during experimental trials.

### Fine needle muscle biopsy procedure

Fine needle muscle biopsies were performed on the vastus lateralis muscle of the participant's left leg using a spring‐loaded, reusable instrument with 14‐gauge disposable needles, and a coaxial introducer (Argon Medical Devices Inc., Plano, TX). Following local anesthesia with 2 mL of 1% lidocaine applied into the subcutaneous tissue, a small incision to the skin was made and an insertion cannula was placed perpendicular to the muscle until the fascia was pierced. The biopsy needle was inserted through the cannula and a muscle sample was obtained by the activation of a trigger button, which unloaded the spring and activated the needle to collect a muscle sample. Multiple biopsy passes at each time point were made with the cannula in place, thus avoiding repeated skin punctures. Each muscle sample was removed from the biopsy needle using a sterile scalpel and was subsequently placed in a cryotube, rapidly frozen in liquid nitrogen, and stored at −80°C. All muscle biopsies were performed by a licensed physician.

### Intramuscular signaling analysis

Tissue samples were thawed and transferred to a conical tube on ice for preparation and homogenization. Each sample was washed with phosphate‐buffered saline (PBS), centrifuged for 1 min at 5000 × g, and excess PBS was subsequently aspirated from the conical tube. A lysis buffer with protease inhibitor (EMD Millipore, Billerica, MA) was then added to each sample at a rate of 500 *μ*l per 10 mg of tissue. Samples were homogenized using a Teflon pestle and sonication (Branson, Danbury, CT). Tissue samples were then placed on a plate shaker (Thermo Fisher Scientific Inc., Waltham, MA) for 10 min at 4°C and subsequently centrifuged at 10,000 × g for 5 min. The supernatant was aspirated and used for analysis.

Multiplex enzyme‐linked immunosorbent assay (ELISA) was used to quantify the phosphorylation status of proteins specific to the Akt/mTOR intracellular signaling pathway using MAGPIX^®^ (Luminex, Austin, TX) and a multiplex Akt/mTOR signaling assay kit (EMD Millipore, Billerica, MA) according to manufacturer's guidelines. Multiplex ELISA has been validated (Hwang 2011) and previously used to determine the phosphorylation status of proteins in the Akt/mTOR signaling pathway (Sharma et al. 2012a,b). Samples were analyzed for phosphorylation of protein kinase B (Akt) at Ser 473, mTOR at Ser 2448, p70S6k at Thr 412, and ribosomal protein S6 (RPS6) at Ser 235/236. Total protein quantification was conducted using a detergent compatible (DC) protein assay kit (Bio‐Rad, Hercules, CA). Homogenized samples were diluted prior to being loaded and results are reported as fluorescence intensity expressed relative to total protein content. To eliminate interassay variance, all tissue samples were thawed once and analyzed in duplicate in the same assay run by a single technician. The average coefficient of variation for phosphoprotein analysis was 5.6%.

We employed the same multiplex platform as previously described by Sharma et al. (2012b). In this study, the authors assessed and reported multiple targets but only validated one target (Akt) by immunoblotting. The multiplex platform utilized in our study, and that of Sharma et al. (2012b), detects Akt at the serine 473 phosphorylation site. Sharma et al. (2012b) detected a positive signal at serine 473 via immunoblotting that confirmed the multiplexing results. Although our multiplex ELISA data lack in‐house cross‐validation with immunoblotting, based on that which has been previously described by Sharma et al. (2012b), we are confident in our results as they agree with a number of other similarly designed studies that have shown similar results with our chosen protein targets using ELISA and immunoblotting (Farnfield et al. 2009; Hulmi et al. 2012; Areta et al. 2013).

### Myosin heavy chain mrna expression

#### Total RNA isolation

Total cellular RNA was extracted from homogenate of biopsy samples (10–15 mg) with a monophasic solution of phenol and guanidine isothiocyanate contained within the TRI‐reagent (Sigma Chemical Co., St. Louis, MO). About 500 *μ*L of TRI‐Reagent was added to each tube, and then muscle samples were homogenized using a pestle. About 100 *μ*L of chloroform was added to each tube and shaken, then allowed to sit for 15 min. This process separated the samples into three distinct phases, a lower organic phase which contains the protein, a middle interphase containing the DNA, and an upper aqueous phase containing the RNA. Using a sterile transfer pipette, the clear aqueous phase was transferred into a new microfuge tube. The remaining interphase and organic phase was stored at −80°C. Subsequently, 250 *μ*L of 100% isopropanol was added to each tube and allowed to sit at room temperature for 5–10 min. Samples were then centrifuged at 12,000 × *g* at 2–8°C for 10 min, allowing for the formation of a RNA pellet. The supernatant was discarded, and 500 *μ*L of 75% ethanol was added and then vortexed to wash the pellet. The samples were then centrifuged at 7500 × *g* at 2–8°C for 5 min then the supernatant was discarded. The washing procedure was repeated twice. The pellet was allowed to air dry for 5–10 min, then 50 *μ*L of nuclease‐free water was added to the microtube. The total RNA concentration was determined spectrophotometerically (SmartSpec Plus, Bio‐Rad) by optical density (OD) at 260 nm using an OD_260_ equivalent to 40 *μ*g · *μ*L^−1^ and the final concentration expressed relative to muscle wet‐weight. Aliquots of total RNA (5 *μ*L) were separated with 1% agarose gel electrophoresis, ethidium bromide stained, and monitored under an ultraviolet light (Chemi‐Doc XRS, Bio‐Rad) to verify RNA integrity and absence of RNA degradation, indicated by prominent 28s and 18s ribosomal RNA bands, as well as an OD_260_/OD_280_ ratio of approximately 2.0. The RNA samples were stored at −80°C until later analysis.

#### Reverse transcription and complementary DNA (cDNA) synthesis

Five microgram of total skeletal muscle RNA was reverse‐transcribed to synthesize cDNA using the iScript cDNA Synthesis Kit (Bio‐Rad). Each reverse transcription reaction mixture was incubated at 25°C for 5 min, 42°C for 30 min, heated to 85°C for 5 min, and then quick‐chilled on ice. The cDNA concentration was determined by using an OD_260_ equivalent to 50 *μ*g·*μ*L^−1^ and starting cDNA template concentration was standardized by adjusting all samples to 200 ng prior to amplification.

#### Oligonucleotide primers for polymerase chain reaction (PCR)

The mRNA sequences of human skeletal muscle *β*‐actin (NM_001101), MHC I (X06976), MHC IIA (AF111784), and MHC IIX (AF111785) published in the NCBI Entrez Nucleotide database (www.ncbi.nlm.nih.gov) were used to construct PCR primers using Beacon Designer software (Bio‐Rad), and then commercially synthesized (Integrated DNA Technologies, Coralville, IA). These primers amplify fragments of 150 base pairs (bp) for MHC I, 145 bp for MHC IIA, and 148 bp for MHC IIX. Due to its consideration as a constitutively expressed “housekeeping gene,” and the fact that it has been shown to be an appropriate external reference standard in human skeletal muscle using real‐time PCR, *β*‐actin was used for detecting the relative change in the quantity of mRNA in response to resistance exercise (Mahoney et al. 2004). For *β*‐actin, these primers amplify a PCR fragment of 135 base pairs.

#### Real‐time PCR amplification and quantitation

Around 2 μL of cDNA template, 12.5 *μ*L of iQ SYBR Green Supermix (Bio‐Rad), 1.5 *μ*L of the reverse primer reaction mixture, 1.5 *μ*L of the forward primer reaction mixture, and 7.5 *μ*L of nuclease‐free water were added to each well. Each reaction was amplified using real‐time quantitative PCR (iCycler IQ Real‐Time PCR Detection System, Bio‐Rad). The amplification profile was run for 40 cycles employing a denaturation step at 95°C for 30 sec, primer annealing at 58°C for 30 sec, and extension at 72°C for 30 sec. Fluorescence was measured after each cycle resulting from the incorporation of SYBR green dye into each amplicon. The expression of mRNA was determined from the ratio of the C_T_ values relative to *β*‐actin. The specificity of the PCR was demonstrated with an absolute negative control reaction containing no cDNA template, and a single gene product was confirmed using DNA melt curve analysis. Positive amplification of the amplicons were assessed with agarose gel electrophoresis illuminated with UV transillumination (Chemi‐Doc XRS, Bio‐Rad).

#### Skeletal muscle MHC I, MHC IIA, and MHC IIX mRNA expression

The mRNA expression of MHC I, MHC IIA, and MHC IIX genes were performed using real‐time PCR based on our previously established guidelines (Willoughby and Nelson 2002). Oligonucleotide primers were designed using Primer Express from known human mRNA sequences available online through the NCBI database. The expression of mRNA was determined relative to the expression of *β*‐actin. The 2^−ΔCT^ method was used to determine basal gene expression, while fold changes following exercise were calculated using the 2^−ΔΔCT^ method (Livak and Schmittgen 2001). The specificity of the PCR was demonstrated with an absolute negative control reaction containing no cDNA template, and single gene products confirmed using DNA melt curve analysis.

### Myosin heavy chain protein isoform quantification

Myosin heavy chain protein isoform quantification analysis was performed on the BL muscle sample of each participant. Total muscle protein content was determined spectrophotometrically based on the Bradford method at a wavelength of 595 nm (Bradford 1976). A standard curve was generated using bovine serum albumin (Bio‐Rad), and based on previous research, myofibrillar protein content was expressed relative to muscle wet‐weight (Shelmadine et al. 2009; Spillane et al. 2009).

Protein concentrations were diluted to 2 mg mL^−1^ and the MHC protein isoform composition was determined under denaturing conditions using an Experion Pro260 automated electrophoresis system (Bio‐Rad) using the principles of SDS‐PAGE and LabChip (Caliper Life Sciences, Hopkinton, MA) technology (Willoughby et al. 2007; Spillane et al. 2009). Gel images were then processed and displayed on a computer monitor and MHC bands identified by migration relative to the molecular weight marker (data not shown). The densities of the MHC bands was determined using Experion Imaging software (Bio‐Rad) and are expressed in arbitrary density units.

### Dietary logs

Participants were instructed to maintain their normal dietary intake leading up to experiment trials. Participants were then instructed to record as accurately as possible everything they consumed during the 24‐h prior to the first experimental trial. For the following experimental trial, participants were required to duplicate the content, quantity, and timing of their daily diet. Participants were instructed not to eat or drink (except water) within 10 h of reporting to the HPL for experimental trials.

### Statistical analysis

Intramuscular signaling and MHC mRNA expression was analyzed using a two factor trial (REX and CTL) × time (BL, 2H, and 6H) analysis of variance (ANOVA) with repeated measures on time. In the event of a significant F ratio, LSD post hoc tests were used for pairwise comparisons. Percent changes from baseline measures were calculated for anabolic signaling, and Pearson's product–moment correlation was used to examine selected bivariate measures. Prior to statistical procedures, all data were assessed for normal distribution, homogeneity of variance, and sphericity. If assumption of sphericity were violated, a Greenhouse‐Geisser correction was applied. Significance was accepted at an alpha level of *P* ≤ 0.05 and all data are reported as mean ± SD.

## Results

### Akt/mTOR signaling

**Figure 1.**
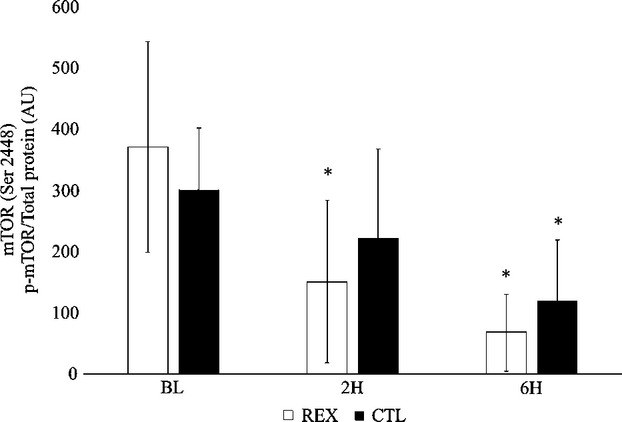
Phosphorylation of mTOR^S^^er2448^. Participants performed a high‐volume resistance exercise protocol (REX) and a control protocol (CTL). Muscle biopsies were performed at baseline (BL), 2 h (2H), and 6 h (6H) post‐exercise. Phosphorylation of mTOR (p‐mTOR) was determined relative to total protein concentration and are therefore reported as arbitrary units (AU). Values are means ± SD. *Significantly different from BL (*P* < 0.05).

No significant differences between trials were noted for phosphorylation of Akt (*F* = 0.95; *P* = 0.40; *η*^2^=0.06) and no significant time effects were noted for phosphorylation of Akt (*F* = 3.15; *P* = 0.18; *η*^2^=0.18). No significant differences between trials were noted for phosphorylation of mTOR (*F* = 2.11; *P* = 0.14; *η*^2^=0.13), however, significant time effects were observed for phosphorylation of mTOR (*F* = 21.65; *P* = 0.0001; *η*^2^=0.61). During REX, phosphorylation of mTOR was significantly decreased from BL at 2H and 6H (*P* = 0.01 and *P* = 0.003, respectively). During CTL, phosphorylation of mTOR was also significantly decreased from BL at 6H (*P* = 0.01) (Fig. 1).

**Figure 2.**
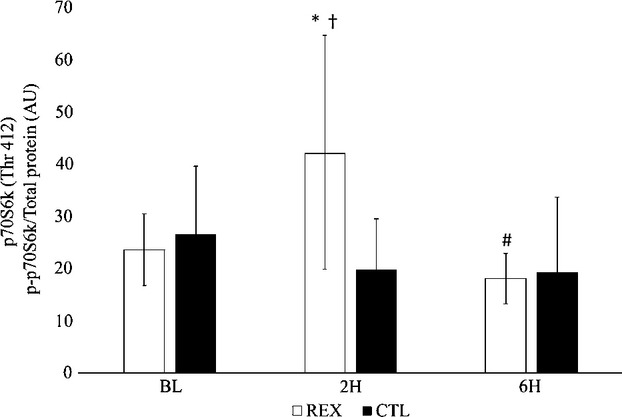
Phosphorylation of p70S6k^Thr412^. Participants performed a high‐volume resistance exercise protocol (REX) and a control protocol (CTL). Muscle biopsies were performed at baseline (BL), 2 h (2H), and 6 h (6H) post‐exercise. Phosphorylation of p70S6k (p‐p70S6k) was determined relative to total protein concentration and are therefore reported as arbitrary units (AU). Values are means ± SD. *Significantly different from BL (*P* < 0.05). ^#^Significantly different from 2H. ^†^Significantly different from CTL within that time point (*P* < 0.05).

Significant differences between trials were noted for phosphorylation of p70S6k (*F* = 3.71; *P* = 0.04; *η*^2^=0.27). Phosphorylation of p70S6k was significantly greater in REX compared to CTL at 2H (*P* = 0.04). Significant time effects were noted for phosphorylation of p70S6k (*F* = 4.61; *P* = 0.02; *η*^2^=0.32). During REX, phosphorylation of p70S6k was significantly elevated from BL at 2H (*P* = 0.03) and returned back to baseline levels by 6H (*P* = 0.02) (Fig. 2).

**Figure 3.**
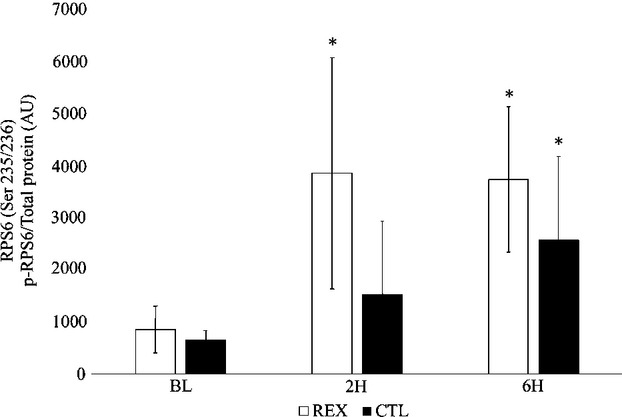
Phosphorylation of RPS6^Ser235/236^. Participants performed a high‐volume resistance exercise protocol (REX) and a control protocol (CTL). Muscle biopsies were performed at baseline (BL), 2 h (2H), and 6 h (6H) post‐exercise. Phosphorylation of RPS6 (p‐RPS6) was determined relative to total protein concentration and are therefore reported as arbitrary units (AU). Values are means ± SD. *Significantly different from BL (*P* < 0.05).

Although no significant differences between trials were noted for phosphorylation of RPS6 (*F* = 2.61; *P* = 0.09; *η*^2^=0.16), a trend was observed in the response between REX and CTL in the phosphorylation of RPS6 at 2H. Significant time effects were noted for phosphorylation of RPS6 (*F* = 14.95; *P* = 0.0001; *η*^2^=0.52). During REX, RPS6 phosphorylation significantly increased from BL at 2H and 6H (*P* = 0.01 and *P* = 0.0001, respectively). During CTL, phosphorylation of RPS6 also significantly increased from BL at 6H (*P* = 0.01) (Fig. 3).

### Myosin heavy chain mRNA expression

No significant differences were noted between trials for type I MHC mRNA expression (*F* = 1.77; *P* = 0.19; *η*^2^=0.12), type IIA MHC mRNA expression (*F* = 0.49; *P* = 0.62; *η*^2^=0.04), or type IIX MHC mRNA expression (*F* = 1.04; *P* = 0.35; *η*^2^=0.07). In addition, no significant time effects were observed for type I MHC mRNA expression (*F* = 0.002; *P* = 0.99; *η*^2^=0.0001), type IIA MHC mRNA expression (*F* = 0.28; *P* = 0.76; *η*^2^=0.02), or type IIX MHC mRNA expression (*F* = 0.12; *P* = 0.82; *η*^2^=0.01) during the postexercise period.

### Association between maximal strength and anabolic signaling

**Figure 4.**
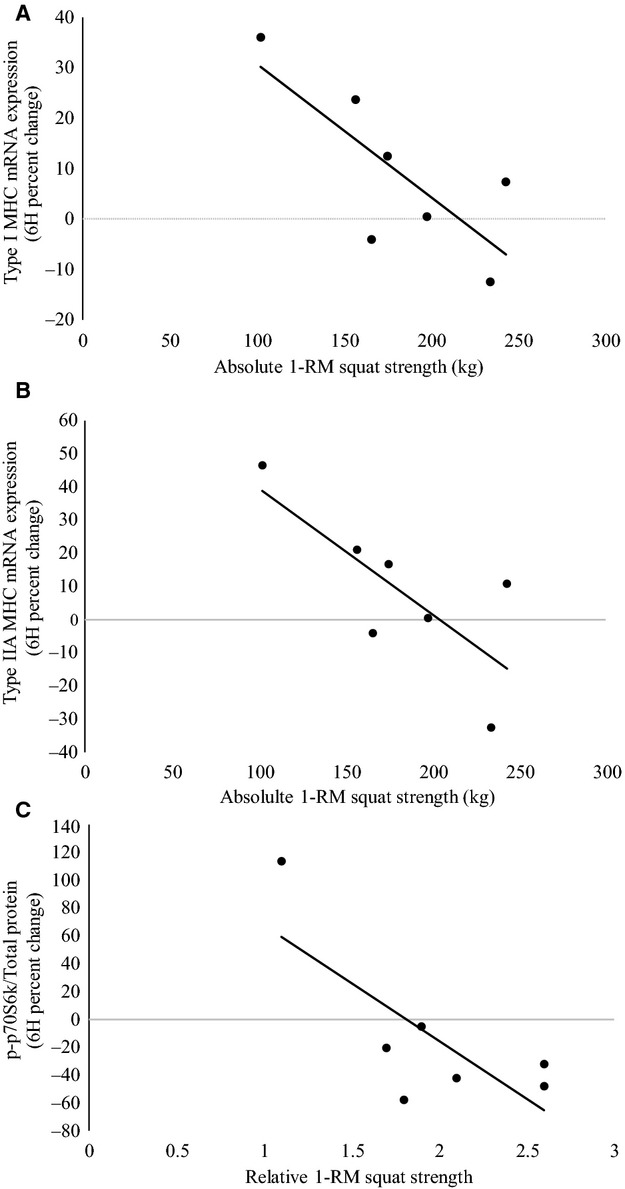
The association between maximal strength and anabolic signaling (A) Correlation between absolute 1‐RM squat strength and type I MHC mRNA expression at 6H (*r* = −0.77; *P* = 0.04) (B) Correlation between absolute 1‐RM squat strength and type IIA MHC mRNA expression at 6H (*r* = −0.75; *P* = 0.05). (C) Correlation between relative 1‐RM squat strength and percent change in p70S6k phosphorylation at 6H (*r* = −0.75; *P* = 0.05).

The association between maximal strength and anabolic signaling are shown in Figure 4. A strong, significant correlation was observed between absolute 1‐RM squat strength and the percentage of type IIX MHC isoform (*r* = 0.89; *P* = 0.003). Absolute 1‐RM squat strength was also inversely correlated with the percent change in type I MHC mRNA expression (*r* = −0.77; *P* = 0.04) and type IIA MHC mRNA expression (*r* = −0.75; *P* = 0.05) at 6H. There was also a significant inverse correlation between relative 1‐RM squat strength and the percent change in p70S6k phosphorylation at 6H (*r* = −0.75; *P* = 0.05).

### Association between MHC protein isoform composition and anabolic signaling

**Figure 5.**
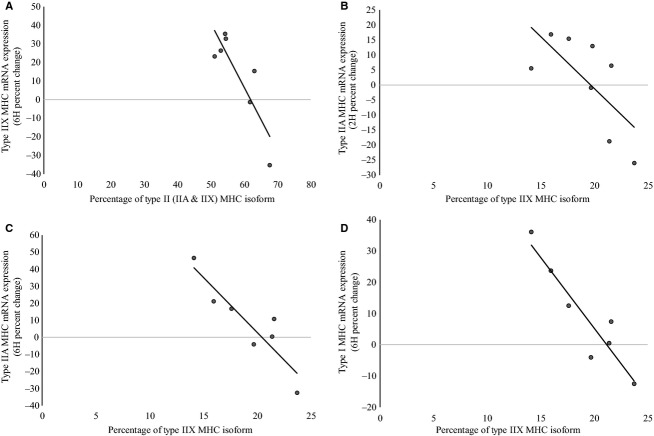
The association between MHC protein isoform composition and anabolic signaling. (A) Correlation between percentage of type II (IIA and IIX) MHC isoforms and percent change in type IIX MHC mRNA expression at 6H (*r* = −0.86; *P* = 0.01). (B) Correlation between percentage of type IIX MHC isoform and percent change in type IIA MHC mRNA expression at 2H (*r* = −0.69; *P* = 0.05) (C) Correlation between percentage of type IIX MHC isoform and and percent change in type IIA MHC mRNA expression at 6H (*r* = −0.90; *P* = 0.01). (D) Correlation between percentage of type IIX MHC isoform and percent change in type I MHC mRNA expression at 6H (*r* = −0.93; *P* = 0.003).

The participants' percentage of type I MHC isoform, type IIA MHC isoform, and type IIX MHC isoform was 42.17 ± 5.77%, 38.59 ± 4.01%, and 19.24 ± 3.17%, respectively. The association between MHC protein isoform composition and anabolic signaling is shown in Figure 5. A strong, significant correlation was observed between the percentage of type I MHC isoform and the percent change in type IIX MHC mRNA expression at 6H (*r* = 0.86; *P* = 0.01). Similarly, the percentage of type II (IIA and IIX) MHC isoforms were inversely correlated with the percent change in type IIX MHC mRNA expression at 6H (*r* = −0.86; *P* = 0.01). The percentage of type IIX MHC isoform was inversely correlated with the percent change in type IIA MHC mRNA expression at 2H (*r* = −0.69; *P* = 0.05) and at 6H (*r* = −0.90; *P* = 0.01). The percentage of type IIX MHC isoform was also inversely correlated with the percent change in type I MHC mRNA expression at 6H (*r* = −0.93; *P* = 0.003).

## Discussion

The biochemical events promoting muscle protein synthesis are regulated by multiple intramuscular signaling mechanisms. Resistance exercise initiates a multifaceted biochemical response regulating mRNA translation. In the current study, signaling proteins within the Akt/mTOR pathway and MHC mRNA expression were examined following a high‐volume, whole body resistance exercise protocol in experienced, resistance‐trained men. The exercise protocol employed was typical to what one may see being used by an athlete focusing on muscle hypertrophy (Kraemer et al. 2002). During the exercise protocol, significantly greater increases in p70S6k phosphorylation were observed compared to control conditions. Increases (102%) from BL in the phosphorylation of p70S6k were seen at 2H, and returned to baseline values by 6H. Significant elevations in RPS6 were seen at both 2H and 6H, while elevations were observed at 6H only during CTL. A trend toward a difference between trials was noted. Considering that amino acid ingestion is a potent stimulus to protein signaling (Beugnet et al. 2003), it is likely that these changes may be related in part to the postexercise meal, and not solely to the exercise stimulus. Akt phosphorylation, mTOR phosphorylation, and MHC mRNA expression did not appear to be altered by the resistance exercise protocol.

The phosphorylation of p70S6k regulates several factors involved in translation initiation and protein synthesis (Goodman 2014), and the phosphorylated state of p70S6k has shown to be a proxy marker of myofibrillar protein synthesis rates (Kumar et al. 2009; West et al. 2010a) and exercise‐induced hypertrophy (Baar and Esser 1999; Terzis et al. 2008; Mayhew et al. 2009; Mitchell et al. 2013). Following resistance exercise, rapid elevation of p70S6k phosphorylation has been observed in untrained and recreationally active men in both fed (Karlsson et al. 2004; Farnfield et al. 2009; Hulmi et al. 2009; Apró and Blomstrand 2010; Deldicque et al. 2010) and fasted (Dreyer et al. 2006, 2010; Terzis et al. 2008; Drummond et al. 2009; Roschel et al. 2011) states. Resistance exercise‐induced elevations of p70S6k have also been observed in experienced, resistance‐trained men (Glover et al. 2008; Areta et al. 2013), yet prior training appears to attenuate the early signaling response (Coffey et al. 2006). The results of this study provide additional support to the rapid exercise‐induced activation of p70S6k in experienced, resistance‐trained men when provided a meal following a resistance exercise session (Farnfield et al. 2009; Hulmi et al. 2009; Deldicque et al. 2010; Moore et al. 2011; Reitelseder et al. 2011; Areta et al. 2013).

RPS6 is a downstream target of p70S6k with the potential to regulate protein synthesis (Goodman 2014) and is commonly used as an indirect marker of mTORC1 activation. Several studies have reported an upregulation of RPS6 phosphorylation following resistance exercise (Drummond et al. 2009; Hulmi et al. 2009, 2012; Apró and Blomstrand 2010). However, the exact role of RPS6 in the regulation of protein synthesis remains to be determined. Although the resistance exercise protocol employed in this study did not result in a significantly greater elevation in RPS6 phosphorylation compared to CTL, a trend was noted for an upregulation of RPS6 phosphorylation at 2H post‐exercise. Interestingly, phosphorylation of the upstream signaling molecules, Akt and mTOR, was not significantly altered by the resistance exercise protocol compared to control conditions.

The rate of MHC protein synthesis has been demonstrated to be associated with the rate of mixed muscle protein synthesis following resistance exercise (Hasten et al. 2000). Exercise‐induced upregulation of MHC mRNA content presumably increases the rate of translational encoding for the respective MHC protein (McCall et al. 1999; Willoughby and Rosene 2001). The results of this study indicated that the acute resistance exercise protocol was unable to influence type I, type IIA, or type IIX MHC mRNA expression at any time point. These results support a previous study that demonstrated an increase in myofibrillar protein synthesis following resistance exercise without altering MHC mRNA concentrations suggesting that exercise‐induced myofibrillar protein synthesis may be mediated by a more efficient translation of mRNA (Welle et al. 1999). An increased concentration of MHC mRNA presumably leads to an increase in the synthesis of the respective MHC protein, however, it is unclear to what extent the magnitude of total MHC mRNA influences changes in overall muscle protein synthesis. In contrast to our findings, Willoughby and Nelson (2002) reported significant increases in type I, type IIA, and type IIX MHC mRNA expression at 6 h following three sets of 8–10 repetitions at 75–80% 1‐RM on the squat, leg press, and leg extension exercises in recreationally active men. These differences may be related to differences in the training experience and strength levels of the participants.

The MHC isoform composition of muscle fibers is a major determinant of the contractile characteristics of skeletal muscle, which governs maximal muscle shortening velocity and maximal power output (Bottinelli et al. 1996, 1999; Stienen et al. 1996). Although muscle fibers may have two or more MHC isoforms, one of these isoforms tends to dominate, leading to a specific fiber type characterization (Larsson and Moss 1993). Consistent with previous investigations (Harridge et al. 1996; Aagaard and Andersen 1998), we observed a relationship between muscle strength and the percentage of the MHC IIX isoform. MHC isoform composition may also impact the genes regulating translation initiation signaling in response to resistance exercise. Exercise‐induced phosphorylation of mTOR has been demonstrated to be specific to fibers predominantly expressing the MHC IIA isoform in rats (Parkington et al. 2003). In addition, exercise‐induced phosphorylation of p70S6k has shown to be significantly greater in type II fibers, compared to type I fibers in untrained men (Koopman et al. 2006), suggesting that this subpopulation of fibers may be more responsive to hypertrophy. In support, Tannerstedt et al. (2009) demonstrated a significant increase in phosphorylation of p70S6k and RPS6 in type II fibers, whereas type I fibers remained unchanged following maximal lengthening contractions in untrained men. To our knowledge, the present study is the first to investigate the association between MHC isoform composition or muscle fiber subtypes and anabolic signaling in experienced, resistance‐trained men. The percentage of type IIX isoform was found to be inversely correlated with type I and type IIA MHC mRNA expression, suggesting that participants with the highest percentages of the type IIX isoform had the lowest type I and type IIA MHC mRNA expression. In addition, strength was inversely correlated with type I and type IIA MHC mRNA expression and p70S6k phosphorylation. In contrast to investigations examining untrained subjects, the results of this study appear to indicate that a greater proportion of type IIX MHC isoform, along with higher levels of strength, will attenuate the acute anabolic response. This suggests a potential lower adaptive ability among highly trained individuals, and may, in part, account for the diminished hypertrophic adaptation among athletes with increased training experience (Häkkinen et al. 1987; Hoffman et al. 1991). These results are also consistent with the findings of Coffey et al. (2006) who reported that prior training history blunts the anabolic signaling responses involved in the adaptation to resistance exercise. Similarly, Tang and colleagues (2008) reported that the duration of protein synthesis following a bout of resistance exercise was attenuated following 8 weeks of resistance training. While fiber type characterization may, in part, explain the large variability in early phase hypertrophic responses in untrained individuals, training experience and percentage of type IIX MHC may contribute to a blunted resistance exercise‐induced anabolic response in the experienced, resistance‐trained athlete.

The results of this study are specific to the Akt/mTOR pathway and MHC mRNA expression following a high‐volume, whole body resistance exercise protocol in experienced, resistance‐trained men. Whether these results are consistent with a different training paradigm is unknown. Additionally, the current study implemented a standardized breakfast, a recovery drink immediately following exercise, and a small meal to simulate what one may see being used by an athlete. Therefore, the synergistic effect of resistance exercise and amino acids must be acknowledged (Apró and Blomstrand 2010). We also recognize that the methods of studying intramuscular signaling in vivo in humans are accompanied by inherent limitations as it requires repeated biopsy sampling of a small population of muscle fibers at a few, distinctive time points following exercise and the analyzed tissue is assumed to be representative of the entire muscle.

In conclusion, the high‐volume resistance exercise protocol resulted in significant increases in p70S6k phosphorylation at 2H post‐exercise compared to control conditions in experienced, resistance‐trained men. MHC mRNA expression and other targets in the Akt/mTOR pathway did not appear to be significantly influenced by the resistance exercise protocol. However, the percentage of type IIX isoform was inversely correlated with type I and type IIA MHC mRNA expression following resistance exercise. Furthermore, strength levels were also demonstrated to be inversely correlated with Type I and Type IIA MHC mRNA expression and p70S6k phosphorylation supporting a potential attenuated hypertrophic response in experienced, resistance‐trained athletes with high levels of strength.

## Acknowledgments

The authors thank Carleigh H. Boone, Gabriel J. Pruna, Gerald T. Mangine, Ran Wang, Amelia A. Miramonti, Michael B. LaMonica, and Mattan W. Hoffman for their assistance in data collection.

## Conflict of Interest

None declared.
